# [Retracted] Apigenin induces apoptosis and counteracts cisplatin-induced chemoresistance via Mcl-1 in ovarian cancer cells

**DOI:** 10.3892/etm.2025.12941

**Published:** 2025-08-11

**Authors:** Yuyan Qi, Zhaoxia Ding, Yushuang Yao, Feifei Ren, Min Yin, Songbin Yang, Aiping Chen

Exp Ther Med 20:1329-1336, 2020; DOI: 10.3892/etm.2020.8880

Following the publication of this paper, it was drawn to the Editors’ attention by a concerned reader that certain of the western blot data featured in Figs. 2, 4 and 6 in this paper were strikingly similar to data in other articles written by different authors at different research institutes that had either already been published, or which were already under consideration for publication, at the time of this paper’s submission to *Experimental and Therapeutic Medicine.* In view of the fact some of the abovementioned data had apparently already been published when this paper was received at *Experimental and Therapeutic Medicine*, the Editor has decided that this paper should be retracted from the Journal. The authors were asked for an explanation to account for these concerns, but the Editorial Office did not receive a reply. The Editor apologizes to the readership for any inconvenience caused.

